# Cardiopulmonary Support During Catheter Ablation of Ventricular Arrhythmias With Hemodynamic Instability: The Role of Inducibility

**DOI:** 10.3389/fcvm.2021.747858

**Published:** 2021-10-20

**Authors:** Massimo Grimaldi, Maria Monica Marino, Nicola Vitulano, Federico Quadrini, Federica Troisi, Nicola Caporusso, Vera Perniciaro, Rosa Caruso, Nicola Duni, Giacomo Cecere, Alberto Martinelli, Pietro Guida, Vito Del Monte, Tommaso Langialonga, Luigi Di Biase, Antonio Di Monaco

**Affiliations:** ^1^Ospedale Generale Regionale “F. Miulli,” Dipartimento di Cardiologia, Bari, Italy; ^2^Policlinico Tor Vergata, Dipartimento di Cardiologia, Rome, Italy; ^3^Ospedale Generale Regionale “F. Miulli,” Dipartimento di Anestesia e Rianimazione, Bari, Italy; ^4^St. David's Medical Center, Texas Cardiac Arrhythmia Institute, Austin, TX, United States; ^5^Montefiore Medical Center, Albert Einstein College of Medicine, Bronx, NY, United States; ^6^Dipartimento di Medicina Clinica e Sperimentale, Universitá di Foggia, Foggia, FG, Italy

**Keywords:** catheter ablation, electrical storm, extracorporeal membrane oxygenation, ventricular arrhythmia, ventricular inducibility

## Abstract

**Background:** Catheter ablation is a treatment option for sustained ventricular tachycardias (VTs) that are refractory to pharmacological treatment; however, patients with fast VT and electrical storm (ES) are at risk for cardiogenic shock. We report our experience using cardiopulmonary support with extracorporeal membrane oxygenation (ECMO) during catheter ablation of VT.

**Methods:** Sixty-two patients (mean age 68 ± 9 years; 94% male) were referred to our center for catheter ablation of repeated episodes of hemodynamically unstable ventricular arrhythmias. ES was defined as the occurrence of three or more VT/ventricular fibrillation episodes requiring electrical cardioversion or defibrillation in a 24-h period. All patients had hemodynamically unstable VTs.

**Results:** Thirty-one patients (group 1) performed catheter ablation without ECMO support and 31 patients (group 2) with ECMO support. At the end of the procedure, ventricular inducibility was not performed in 16 patients of group 1 (52%) due to significant hemodynamic instability. Ventricular inducibility was performed in the other 15 patients (48%); polymorphic VTs were inducible in eight patients. In group 2, VTs were not inducible in 29 patients (93%); polymorphic VTs were inducible in two patients. The median follow-up duration was 24 months. Four patients of group 1 (13%) and five patients of group 2 (16%) died due to refractory heart failure. An implantable cardioverter-defibrillator intervention (shock or antitachycardia pacing) was documented in 13 patients of group 1 (42%) and six patients of group 2 (19%).

**Conclusions:** Extracorporeal membrane oxygenation support during catheter ablation for hemodynamically unstable VTs is a useful tool to prevent acute procedural heart failure and to reduce arrhythmic burden.

## Introduction

Electrical storm (ES) is a life-threatening syndrome that consists of repeated episodes of ventricular tachycardia (VT) or ventricular fibrillation (VF) occurring over a short period of time. Termination of ES requires an appropriate external or implantable cardioverter-defibrillator (ICD) intervention (1). Previous studies have described poor outcomes associated with ES as well as an up to 3-fold increased risk of mortality in patients with ES (1–3).

Catheter ablation for ES can reduce recurrent episodes of ventricular arrhythmias (VAs) and improve patient prognoses (4–7); however, patients with hemodynamically unstable VAs have a rate of procedural complications and mortality. Recent data suggest that cardiopulmonary support with extracorporeal membrane oxygenation (ECMO) can provide valuable support during catheter ablation procedures in this setting (8–14). In particular, the ECMO system is useful for managing intraoperative acute hemodynamic decompensation and can facilitate the accurate mapping and ablation of unstable VAs.

In this study, we report our experience regarding the ablation of hemodynamically unstable VAs with or without ECMO support.

## Methods

### Study Population

A total of 62 patients (mean age 68 ± 9 years; 94% male) were referred to Miulli Hospital for catheter ablation of repeated episodes of hemodynamically unstable sustained VAs between January 2015 and December 2019. All patients had ES and hemodynamically unstable VAs symptomatic for syncope or presyncope. ES was defined as the occurrence of three or more VT/VF episodes requiring electrical cardioversion or defibrillation in a 24-h period (1). All arrhythmias were unresponsive to amiodarone antiarrhythmic therapy. ECMO support was available at our center from January 2017. Thirty-one patients (group 1) performed catheter ablation without ECMO support (from January 2015 to December 2016) and 31 patients (group 2) with ECMO support (from January 2017 to December 2019).

All procedures were performed by expert operators; the surgical team consisted of two electrophysiologists, one interventional cardiologist, one anesthesiologist, two perfusion technicians, and two nurses. Two vascular surgeons were also present if a patient required femoral artery isolation. All patients underwent preoperative Doppler ultrasound and/or angiocomputed tomography of the lower leg to assess the femoral arteries. The study was approved by the local Ethics Committee.

### Electrophysiological Study and Ablation With ECMO Support

All procedures were initiated under conscious sedation with an intravenous infusion of diazepam (10 mg) and fentanyl (0.2 mg) under the supervision of an anesthesiologist; general anesthesia was administered at the discretion of the anesthesiologist. Antibiotic prophylaxis (cefazolin 2 g and teicoplanin 400–600 mg) was administered immediately before the procedure. Intra-arterial blood pressure monitoring and digital pulse oximetry were monitored continuously during the procedure and ICD therapies were inactivated for the duration of the procedure.

In group 2 patients, before inserting ablation catheters into the heart, cannulas for ECMO support were positioned under the supervision of two expert perfusionists. The circuit (Cardiohelp System, Maquet, Rastatt, Germany) consisted of a centrifugal pump, polymethylpentene gas exchanger, heat exchanger, tubing, and variously sized cannulas for venous and arterial cannulation. The appropriate cannula sizes were selected based on an evaluation of vascular diameter from Doppler ultrasound or angiocomputed tomography of the lower leg and on patient weight. The left femoral artery was cannulated and a guidewire was positioned in the right femoral artery. Angiography was performed to visualize the right common femoral artery and the artery was cannulated under fluoroscopic guidance using the Seldinger technique. Two Perclose Proglide (Abbot, North Chicago, United States) suture-mediated closure systems were positioned in the femoral arteries to facilitate closure of the arteries at the end of the procedure. The right femoral vein was also cannulated. The arterial cannula was inserted into the common femoral artery and advanced up to the iliac artery. The venous cannula was advanced up to the right atrium under fluoroscopic guidance. In patients with a small right femoral artery, a small sheath was placed in the superficial femoral artery to permit distal flow and prevent limb ischemia. In three patients with significant femoral arterial atherosclerosis, the ECMO cannulas were positioned by vascular surgeons. ECMO support was started at 3 L and adjusted following the patient's hemodynamics. During support, heparin was administered to a target-activated clotting time of 300 s in cases of endocardial left VA or 250 s in cases of endocardial right or epicardial VA.

In patients with VA of suspected epicardial origin, a pericardial approach was guaranteed before positioning the ECMO circuit. This workflow was adopted so that the pericardial approach was performed before heparin administration. We performed the pericardial approach as described previously (15) and placed a steerable sheath (Agilis, St. Jude Medical) in the pericardial space to allow catheter stability and maneuverability.

In all patients, the ablation catheter was inserted through the femoral artery/vein and located inside of the left/right ventricle or epicardium through the pericardial sheath. Mapping and ablation were performed with a 3.5-mm irrigated catheter with a contact force sensor (Thermocool Smartouch Surround Flow, Biosense Webster, CA, USA) and a three-dimensional mapping system (CARTO, Biosense Webster, Inc., CA, USA). Substrate maps were obtained using a multipolar mapping catheter (Pentaray; Biosense Webster, CA, USA) in 27 patients.

At first, a geometry of the chamber of interest was created using the Thermocool Smartouch Surround Flow or Pentaray catheters (Biosense Webster, CA, USA); then, a substrate map was acquired during sinus rhythm or right ventricular pacing in pacing-dependent patients. In those patients with cardiac resynchronization therapy devices, left ventricular pacing was turned off.

Initially, an accurate substrate ablation was performed targeting the areas of local abnormal ventricular activity and late potentials (16, 17). In patients with a spontaneous induction of clinical VTs during substrate mapping or ablation, activation mapping was attempted if hemodynamic stability. Activation and entrainment mapping were performed to identify critical sites of the VT reentrant circuit as previously described (18–20). In particular, the window of interest was opened from the termination of the first QRS to the onset of the second QRS of the VT cycle, to define the diastolic interval. The last step was ventricular inducibility. Programmed ventricular stimulation after ablation was performed at the right ventricular apex (basal drive 600/500/400 ms up to three extra stimuli). In the case of inducible VTs, a new mapping and ablation was performed until the non-inducibility was obtained.

Extracorporeal membrane oxygenation (ECMO) support was increased during acute hemodynamic decompensation due to spontaneous or induced VT to permit optimal mapping and ablation of arrhythmias. Radiofrequency energy was delivered with a maximum power of 45 W and a targeted impedance decrease of at least 10% from baseline. Ablation was delivered when the contact force was between 7 and 30 g; when the contact force was > 20 g, we used a maximum power of 35 W. Procedural success was defined as an inability to induce sustained VTs and the disappearance of frequent spontaneous premature ventricular complexes.

In all the patients of group 2, the arterial and venous cannulas were removed at the end of the procedure. The right femoral artery was closed using the previously positioned Perclose Proglide closure system and the vein was closed with manual compression. In three patients, ECMO cannulas were removed by vascular surgeons. After ECMO cannula removal, right femoral artery angiography was performed to exclude the possibility of procedural damage (the angiography catheter was inserted through the left femoral artery). Blood inside of the circuit was recovered using an autologous blood recovery machine (Cell Saver®5+, Haemonetics Corporation) and infused into the patient.

Clinical follow-up was performed every month after catheter ablation for the first year than every 3 months. Clinical recurrence, ICD therapy, and procedural complications were recorded for all patients.

### Statistical Analysis

Descriptive statistics are summarized as the mean ± SD for continuous variables and the number or percentages for categorical variables. The Kaplan–Meier method was used to show graphically survival postprocedure. Statistical analyses were performed using STATA software version 14 (Stata, College Station, TX, USA).

## Results

### General Findings and Procedural Data

The main clinical characteristics of patients are reported in Table 1. There were no significant differences regarding most clinical characteristics parameters except for hypertension, coronary artery bypass graft surgery, and pharmacological therapy (antiplatelets, anticoagulants, and statins). No significant differences were found regarding PAINESD risk score, previous catheter ablations for VT, mean length cycle of VTs, and mean left ventricular ejection fraction (Table 1). All patients were refractory to treatment with at least one antiarrhythmic drug. None of the patients presented with ES in the context of acute myocardial infarction. Reversible ischemia was excluded by left heart catheterization in each patient the same day of the procedure or the day before the procedure. None of the patients exhibited alterations in serum electrolytes.

**Table 1:** Patient characteristics by study groups.

	**All**	**No ECMO**	**ECMO**	
	***n*** **= 62**	***n*** **= 31**	***n*** **= 31**	***p***
Age (years)	68 ± 9	69 ± 8	66 ± 10	0.135
Male sex	58 (94%)	30 (97%)	28 (90%)	0.612
Smoking history	33 (53%)	19 (61%)	14 (45%)	0.203
Body mass index (Kg/m^2^)	25.9 ± 3.4	26.6 ± 3.8	25.2 ± 2.8	0.085
Body mass index >30 Kg/m^2^	6 (10%)	5 (16%)	1 (3%)	0.195
Hypertension	38 (61%)	23 (74%)	15 (48%)	0.037
Dyslipidemia	42 (68%)	24 (77%)	18 (58%)	0.103
Diabetes mellitus	15 (24%)	9 (29%)	6 (19%)	0.374
Chronic renal failure	14 (23%)	6 (19%)	8 (26%)	0.544
Familiarity for cardiovascular disease	25 (40%)	15 (48%)	10 (32%)	0.196
Chronic obstructive pulmonary disease	33 (53%)	18 (58%)	15 (48%)	0.445
Dysthyroidism	15 (24%)	8 (26%)	7 (23%)	0.767
Coronary artery disease	47 (76%)	25 (81%)	22 (71%)	0.374
Percutaneous coronary intervention	37 (60%)	18 (58%)	19 (61%)	0.796
Coronary artery bypass graft surgery	14 (23%)	11 (35%)	3 (10%)	0.015
Valve surgery	2 (3%)	2 (6%)	0 (0%)	0.492
NYHA classification III-IV	47 (76%)	23 (74%)	24 (77%)	0.767
Stroke	3 (5%)	1 (3%)	2 (6%)	1.000
Hypertrophic cardiomyopathy	1 (2%)	0 (0%)	1 (3%)	1.000
Idiopathic ventricular fibrillation	2 (3%)	0 (0%)	2 (6%)	0.492
Idiopathic dilated cardiomyopathy	10 (16%)	3 (10%)	7 (23%)	0.167
ARVD	1 (2%)	1 (3%)	0 (0%)	1.000
Previous catheter ablation for VT	15 (24%)	9 (29%)	6 (19%)	0.374
Number of shock/ATP	6.8 ± 2.6	5.7 ± 2.8	7.5 ± 2.3	0.055
Mean length cycle of VT (m)	296 ± 32	285 ± 34	307 ± 30	0.16
Left ventricular ejection fraction (%)	30 ± 9	30 ± 8	30 ± 10	1.000
Left ventricular ejection fraction <25%	32 (52%)	14 (45%)	18 (58%)	0.309
Left ventricular diameter (mm)	61 ± 7	60 ± 5	62 ± 8	0.233
PAINESD risk score	21 ± 6	22 ± 5	21 ± 7	0.373
ACE-inhibitors	26 (42%)	13 (42%)	13 (42%)	1.000
Angiotensin-II-receptor antagonists	12 (19%)	6 (19%)	6 (19%)	1.000
Sacubitril plus valsartan	19 (31%)	10 (32%)	9 (29%)	0.783
Beta-blocker	61 (98%)	31 (100%)	30 (97%)	1.000
Diuretics	52 (84%)	26 (84%)	26 (84%)	1.000
Calcium channel blocker	7 (11%)	5 (16%)	2 (6%)	0.425
Antiplatelet	40 (65%)	24 (77%)	16 (52%)	0.034
Anticoagulant	26 (42%)	7 (23%)	19 (61%)	0.002
Amiodarone	57 (92%)	30 (97%)	27 (87%)	0.354
Statins	41 (66%)	25 (81%)	16 (52%)	0.016
Tapazole	6 (10%)	3 (10%)	3 (10%)	1.000
Levotiroxin	10 (16%)	6 (19%)	4 (13%)	0.490

*Mean ± SD or absolute frequency (percentage). ACE, angiotensin converting enzyme; ARVD, arrhythmogenic right ventricular dysplasia; ATP, antitachycardia pacing; NYHA, New York Heart Association; VT, ventricular tachycardia*.

### Electrophysiological Study and Ablation With ECMO Support

Two patients of group 2 required urgent ECMO support for acute heart decompensation, the other 29 patients underwent the ablation with prophylactic ECMO support considering the hemodynamically unstable VAs. General anesthesia was used in 14 (45%) patients of group 1 and 16 (51%) patients of group 2. We performed epicardial access without complications in one patient of group 1 (3%) and three patients of group 2 (10%); in all these patients, epicardial ablation was performed without complications. In three cases of significant femoral arterial atherosclerosis, ECMO cannulas were positioned by vascular surgeons. In all patients of group 2, the diameters of the arterial and venous cannulas were 15 and 25 Fr, respectively.

The left ventricle was mapped in all patients of group 1. In 21 patients of group 1 (68%), spontaneous clinical VTs were documented during substrate mapping. All VTs were not mapped and they were interrupted with electrical cardioversion due to hemodynamic instability. A median of three electrical shock applications (range 1–5) was used per patient. At the end of substrate ablation, the programmed ventricular stimulation was not performed in 16 patients (52%) due to significant hemodynamic instability. Ventricular inducibility was performed in the other 15 patients (48%); polymorphic VTs were inducible in eight patients and the operator decided to stop the procedure for the hemodynamic instability.

**Figure 1:**
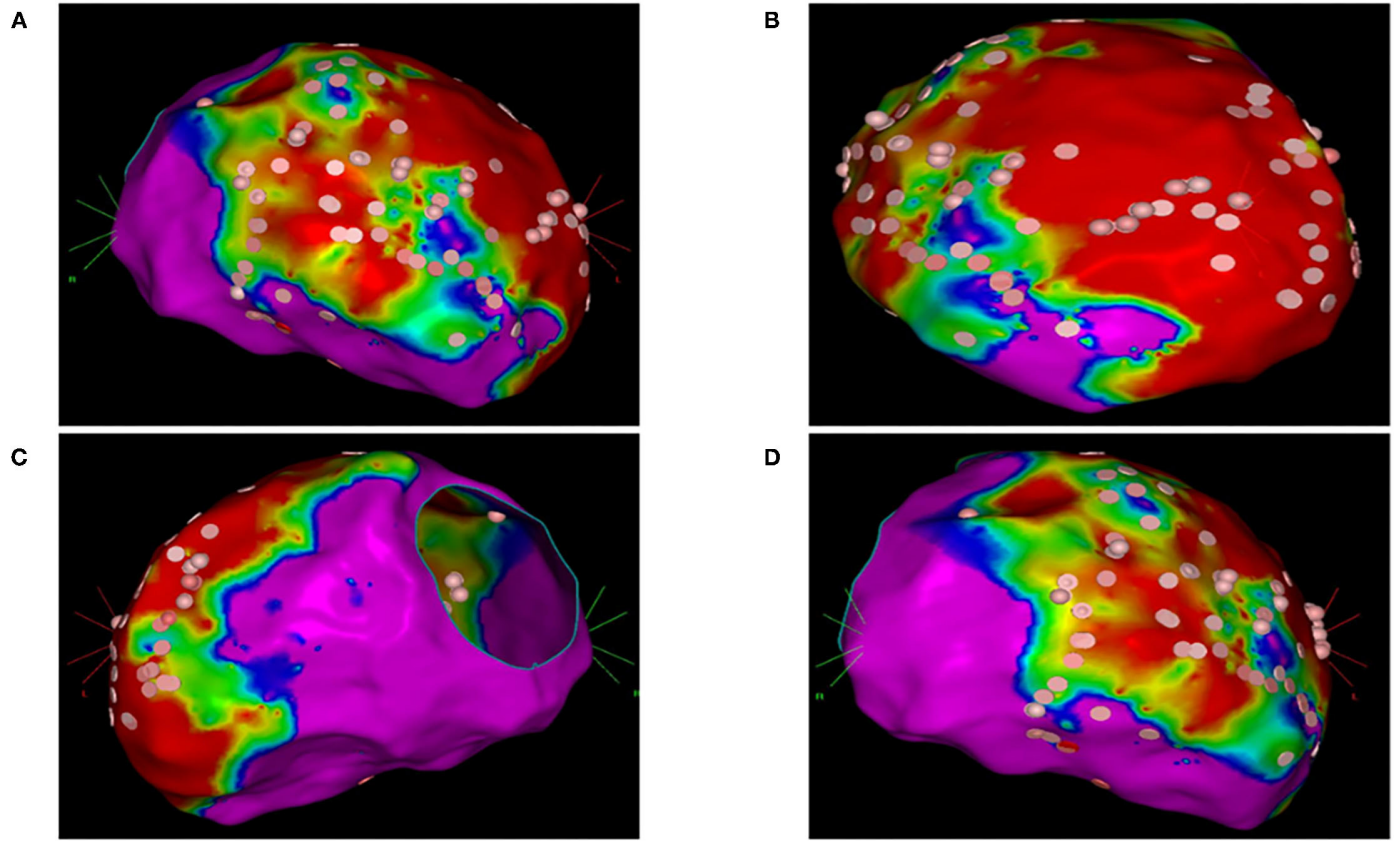
Electroanatomic mapping and ablation of the left ventricle using the CARTO system. **(A)** Anterior-posterior view. **(B)** Left anterior oblique view. **(C)** Left lateral view. **(D)** Right anterior oblique view. Areas with normal voltage (>1.5 mV) are represented with purple color. Areas with low voltage (>0.5 mV) are represented with red color. Border zone areas are represented with blue and green colors (between 0.5 and 1.5 mV). Pink tags represent areas exposed to radiofrequency ablation.

In group 2, the left ventricle was mapped in all patients and the right ventricle was mapped in five patients. In 23 patients of group 2 (74%), 37 VTs induced during substrate mapping were successfully mapped and ablated in all patients. A median of two VTs (range, 1–3) was targeted per patient. ECMO support preserved hemodynamic stability during all VTs permitting an accurate mapping. After substrate ablation, VTs were not inducible in 20 patients of group 2 (64%). In nine patients, the induced VTs were successfully mapped and ablated thus obtaining the absence of ventricular inducibility at the end of the procedure. Polymorphic VTs were inducible in two patients of group 2 and the operator decided to stop the procedure after a mean time of 3.4 h. Among these two patients, electrocardiographic analysis of QRS morphologies was consistent with an epicardial origin of VTs and the operator did not get the pericardial access due to a previous coronary artery bypass graft. ECMO support was removed at the end of the procedure in 30 patients (97%); one patient required long-term ECMO support and died due to refractory heart failure after 5 days. An accurate substrate mapping and ablation was performed in all patients without procedural differences in the two study groups. The mean duration of the ablation procedure and fluoroscopy time were significantly higher in group 2 than in group 1 (Table 2). Figure 1 shows the electroanatomic mapping and ablation in a patient of group 2.

**Table 2:** Procedural data by thre study groups.

	**All**	**No ECMO**	**ECMO**	
	***n*** **= 62**	***n*** **= 31**	***n*** **= 31**	***p***
Total procedure duration (min)	200 ± 68	198 ± 74	201 ± 62	0.882
Fluoroscopy times (min)	8 ± 6	5 ± 3	12 ± 10	<0.001
Dose area product (Gy*cm^2^)	34 ± 46	13 ± 17	55 ± 56	<0.001
Radiofrequency time (min)	49 ± 18	49 ± 18	48 ± 17	0.83
Periprocedural complications	2 (3%)	0 (0%)	2 (6%)	0.492
Epicardial ablation	4 (6%)	1 (3%)	3 (10%)	0.612

*Mean ± SD or absolute frequency (percentage)*.

### Procedural Complications

In group 1, five patients had femoral artery damage (three pseudoaneurysms resolved with arterial compression and two dissections resolved with surgery). In group 2, one patient had the dislodgment of ECMO arterial cannula with hemorrhagic shock and recovered without sequelae, three patients had femoral artery dissection treated by percutaneous angioplasty with stenting.

### Follow-Up

The median follow-up duration was 25 months in group 1 and 24 months in group 2 (range 6–36 months). During follow-up, four patients of group 1 (13%) and five patients of group 2 (16%) died due to refractory heart failure (*p* = 0.45). Furthermore, one patient of group 1 (3%) and one patient of group 2 (3%) died from treatment-refractory ES. The patients with ES recurrence had a VT inducible at the end of the procedure. At final follow-up, an ICD intervention for sustained VT (shock or antitachycardia pacing) was documented in 13 patients of group 1 (42%) and six patients of group 2 (19%) (*p* = 0.017). In both groups, an ICD intervention was documented in all patients with the inducibility of VTs at the end of the procedure. Furthermore, in patients without inducibility of VTs at the end of the procedure (seven patients in group 1 and 29 patients in group 2), an ICD intervention was documented in one patient of group 1 (14%) and four patients of group 2 (13%).

**Figure 2:**
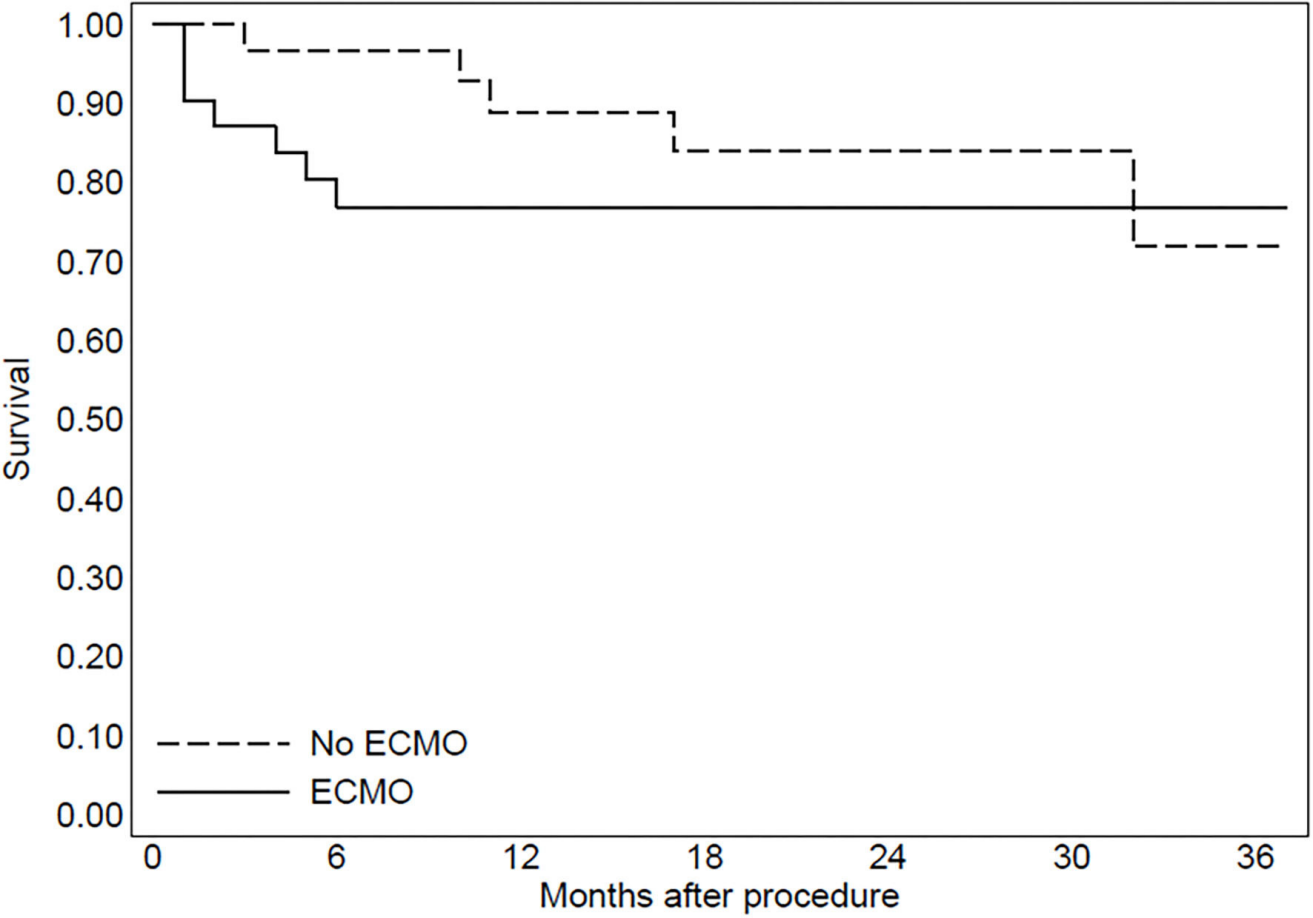
The Kaplan-Meier curve to evaluate mortality due to refractory heart failure after the procedure.

**Figure 3:**
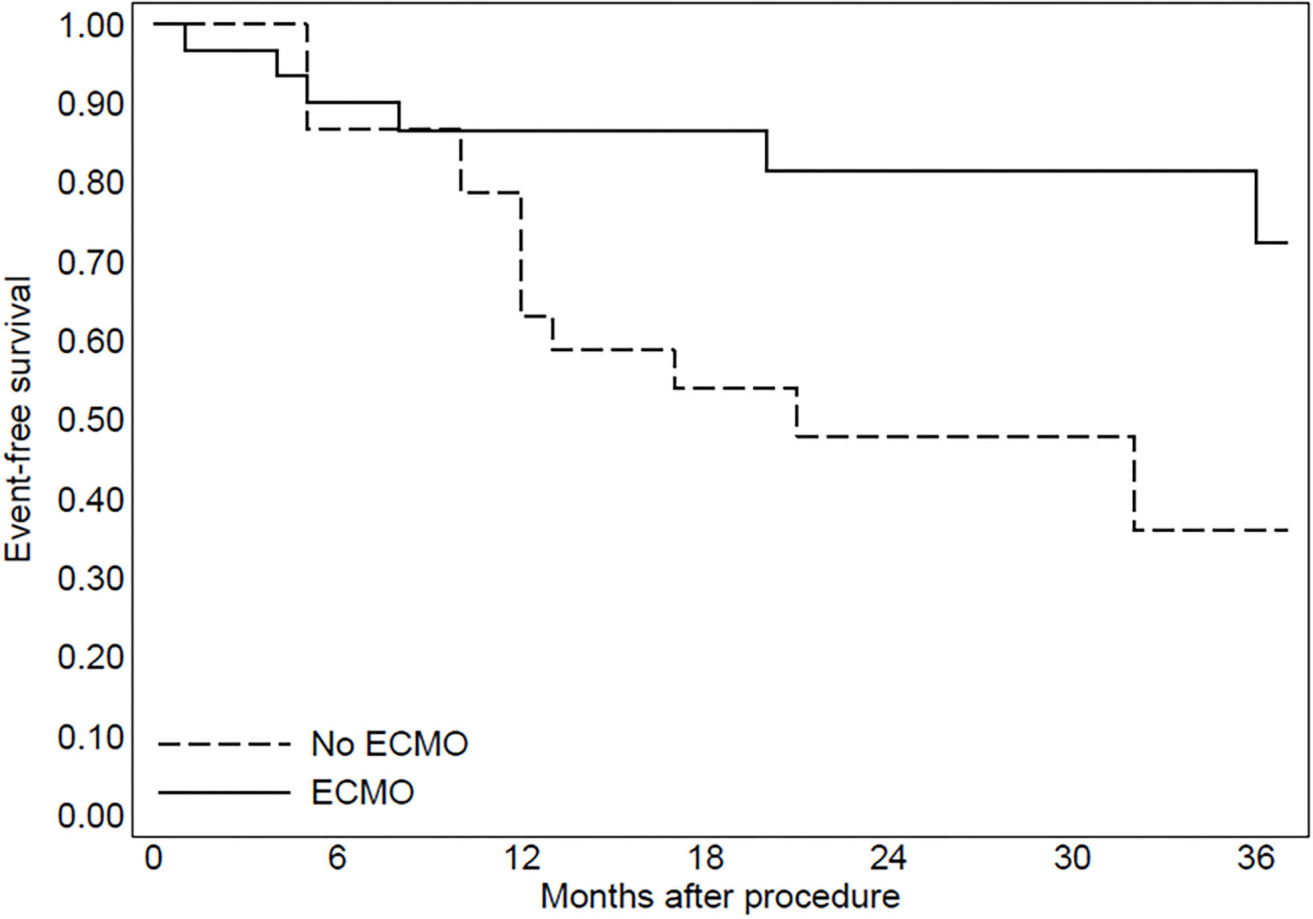
The Kaplan-Meier curve to evaluate ICD interventions after the procedure.

In group 2, three patients were implanted with left ventricular assist devices and one patient underwent heart transplantation. The Kaplan–Meier curve regarding mortality for refractory heart failure and ICD interventions was reported in Figures 2, 3.

## Discussion

The findings of this study indicate that ECMO support during catheter ablation for ES and hemodynamically unstable VTs is a useful tool to prevent acute procedural heart failure and to reduce arrhythmic burden during follow-up.

Catheter ablation is an important treatment option that can achieve VT/VF suppression and provide long-term arrhythmia control in patients with treatment-refractory ES and fast VA (1, 4–7). However, catheter ablation procedures have a high risk of complications: fast VA induced by radiofrequency delivery, catheter movements and pacing maneuvers, multiple electrical cardioversions, and fluid overload can lead to cardiac stunning and acute hemodynamic decompensation.

Most published articles investigated outcomes of emergent cardiopulmonary support with ECMO to rescue acute heart decompensation in patients undergoing catheter ablation of ES showing that ECMO support was associated with poor outcomes when used as a rescue intervention for acute heart decompensation despite hemodynamic stabilization and effective acute arrhythmia suppression (8, 10–14). In particular, the majority of patients studied died of refractory heart failure in the short-term follow-up.

In a recent article (9), we reported data of patients who presented with ES and hemodynamically unstable VTs showing that preemptive ECMO support for patients with ES and hemodynamically unstable VTs was useful to prevent acute heart failure and to reduce procedural complications. A recent article, moreover, reported that the preemptive use of ECMO support for high-risk patients undergoing catheter ablation for VT storm was found to be effective in maintaining hemodynamic status and allowing successful mapping and catheter ablation for VT (14).

In this study, we showed that patients ablated with ECMO support had a reduction in the arrhythmic burden at long-term follow-up. This finding is probably related to the ability in performing safe ventricular inducibility at the end of the procedure without the risk of acute heart failure due to pacing or VT induction. Previous studies, in fact, reported that non-inducibility of VT at the end of the procedure was associated with reduced recurrences during follow-up (21, 22). Recent data reported that the adoption of an extensive induction protocol improved prognosis after VT ablation (23). In particular, patients who were deemed non-inducible for any VT with an extensive induction protocol after the final ablation (up to four extra stimuli ± burst pacing) had a better prognosis compared to patients who were deemed non-inducible for any VT after a limited induction protocol (three extra stimuli). Ventricular inducibility is generally limited in patients with a poor hemodynamic state for the risk of acute heart failure due to pacing and VT induction. Our data, for the first time, reported that ECMO support is useful to perform a safe VT inducibility after ablation avoiding acute heart failure in this population of frail patients.

Furthermore, previous studies investigated the best ablation strategy in patients with unstable VTs. Substrate ablation approach in sinus rhythm is a good strategy of VT ablation but previous data reported that in some cases the hemodynamic support was required because of persistent induction of unstable VTs (12). One study by Bunch et al. (24) provided evidence that hemodynamic support might allow activation mapping of VT with comparable outcomes and complications to an exclusive substrate mapping in sinus rhythm. A meta-analysis including six retrospective observational studies showed that activation/entrainment-guided ablation strategy and substrate-based ablation strategy had similar acute results, long-term outcome and complications rate (25). These results support our hypothesis that in the ECMO group the reduction of arrhythmic burden was not related to the ablation strategy but to the possibility to obtain a non-inducibility of VTs at the end of the procedure.

Finally, no significant reduction of mortality for refractory heart failure was found in patients ablated with ECMO support. Previous studies correlated the reduction of arrhythmic burden to a better survival (26) but the lack of this data in our study is probably because the study population was composed of critical patients with severe heart failure and a worse prognosis. ECMO support, however, improved the quality of life of these patients reducing shock interventions during long-term Follow-up.

The major limitations to this study are the relatively small number of patients and the lack of a randomized design. Another limitation is the low number of patients performing epicardial ablation. Finally, although the procedures were performed by experienced operators, results may have been influenced by the development of mapping and catheter technology over time.

## Conclusion

In this experience, ECMO support facilitated mapping and ablation in patients with ES and hemodynamic instability. The safety in performing ventricular inducibility after ablation is probably the explaining of reduced arrhythmic burden in patients ablated with the ECMO support.

## Data Availability Statement

The raw data supporting the conclusions of this article will be made available by the authors, without undue reservation.

## Ethics Statement

The studies involving human participants were reviewed and approved by Comitato Etico Policlinico Di Bari. Written informed consent for participation was not required for this study in accordance with the national legislation and the institutional requirements.

## Author Contributions

AD, MG, and PG contributed to conception and design of the study. MM organized the database. PG performed the statistical analysis. AD wrote the first draft of the manuscript. NV, FQ, FT, NC, VP, RC, ND, GC, and AM wrote sections of the manuscript. VD, TL, and LD revised the manuscript. All authors contributed to manuscript revision, read, and approved the submitted version.

## Conflict of Interest

The authors declare that the research was conducted in the absence of any commercial or financial relationships that could be construed as a potential conflict of interest.

## Publisher's Note

All claims expressed in this article are solely those of the authors and do not necessarily represent those of their affiliated organizations, or those of the publisher, the editors and the reviewers. Any product that may be evaluated in this article, or claim that may be made by its manufacturer, is not guaranteed or endorsed by the publisher.
